# Evaluation of Monkeypox- and Vaccinia virus-neutralizing antibodies in human serum samples after vaccination and natural infection

**DOI:** 10.3389/fpubh.2023.1195674

**Published:** 2023-06-21

**Authors:** Alessandro Manenti, Niccolò Solfanelli, Paolo Cantaloni, Livia Mazzini, Margherita Leonardi, Linda Benincasa, Giulia Piccini, Serena Marchi, Martina Boncioli, Chiara Spertilli Raffaelli, Danilo Tacconi, Giada Mattiuzzo, Otfried Kistner, Emanuele Montomoli, Claudia Maria Trombetta

**Affiliations:** ^1^VisMederi Srl, Siena, Italy; ^2^VisMederi Research Srl, Siena, Italy; ^3^Department of Molecular and Developmental Medicine, University of Siena, Siena, Italy; ^4^Department of Infectious Diseases, Ospedale San Donato, Arezzo, Italy; ^5^Medicines and Healthcare Products Regulatory Agency, South Mimms, United Kingdom

**Keywords:** humoral immunity, neutralization, Monkeypox virus, vaccinia virus, epidemiology

## Abstract

**Introduction:**

In early to mid-2022, an unexpected outbreak of Monkeypox virus infections occurred outside the African endemic regions. Vaccines originally developed in the past to protect against smallpox are one of the available countermeasures to prevent and protect against *Orthopoxvirus* infections. To date, there are few studies on the cross-reactivity of neutralizing antibodies elicited by previous vaccinia virus-based vaccination and/or Monkeypox virus infection. The aim of this study was to evaluate a possible approach to performing Monkeypox and vaccinia live-virus microneutralization assays in which the read-out is based on the production of cytopathic effect in the cell monolayer.

**Methods:**

Given the complexity of Orthopoxviruses, the microneutralization assay was performed in such a way as to uncover a potential role of complement, with and without the addition of an external source of Baby Rabbit Complement. A set of human serum samples from individuals who had been naturally infected with Monkeypox virus and individuals who may have and not have undergone vaccinia virus vaccinations, was used to evaluate the performance, sensitivity, and specificity of the assay.

**Results and conclusions:**

The results of the present study confirm the presence and cross-reactivity of antibodies elicited by vaccinia-based vaccines, which proved able to neutralize the Monkeypox virus in the presence of an external source of complement.

## 1. Introduction

The genus *Orthopoxvirus* belongs to the *Poxviridae* family. Poxviruses, including Monkeypox virus (MPXV), are large, enveloped viruses consisting of double-stranded DNA of approximately 200 Kbp; they have a diameter of up to 400 nm and code for about 190 genes (1). Upon infection, the viral cycle occurs in the cytoplasm of infected cells; unlike most DNA viruses, poxviruses can complete their life cycle within the cell cytoplasm without invading the nucleus (2, 3). Two infectious viral particles are then produced: an intracellular form, called Intracellular Mature Virus (IMV), with a single membrane embedded with more than 20 antigens, and an extracellular form, called Extracellular Enveloped Virus (EEV), which is coated with an additional outer membrane bearing 8 proteins. The IMV is mainly responsible for host-to-host transmission and is released after cell lysis; the EEV form, upon the budding process, is responsible for cell-to-cell transmission (4). Various surface antigens have proved to be essential to the attachment/entry of the virus to the target cells, and antibodies against many of these are able to neutralize the virus in a complement-dependent manner (5). Although additional studies are needed in order to completely understand the specific mechanisms of binding, the first contact between the host cell and the IMV is mediated by the binding of glycosaminoglycans and heparin sulfate oligosaccharides to the A29 and L1R surface proteins (6). Along with MPXV, the genus *Orthopoxvirus* comprises smallpox virus, vaccinia virus (VACV), rabbitpox virus, cowpox virus, camelpox virus and other strains recently isolated from humans and animals. MPXV was first isolated in 1958 in Denmark after two outbreaks in cynomologus macaques imported from Singapore (7). MPXV is the aetiological agent responsible for a contagious zoonotic disease affecting humans, now called mpox according to a recent World Health Organization (WHO) recommendation (8). Its symptoms include tiredness, fever, rash, variable respiratory distress and muscle pain. A vesiculopustular rash may appear on the face and some parts of the trunk before spreading to other peripheral parts of the body (9). Over several decades, MPXV infections in humans were mainly reported in Central and Western Africa (10, 11), with occasional cases reported in the United States of America (US), presumably caused by infected prairie dogs, and the United Kingdom, linked to international travels (12–14). A new level of threat was reached in April–May 2022, when mpox cases arose in Europe and the US in people who had not traveled to endemic areas and had no contact with persons coming from mpox-endemic countries. This prompted the Director-General of the WHO to declare the seventh Public Health Emergency of International Concern (PHEIC) in July 2022 (15, 16). Interestingly, from 2022 onwards, MPXV isolates showed a higher mutation rate than had formerly been observed (17). Although mpox can be considered to be less severe than smallpox, the outbreak has already resulted in more than 86,000 confirmed cases in more than 110 countries (18, 19). Earlier experience with vaccines against smallpox indicated that the antibodies induced by smallpox vaccines have the potential to cross-react with MPXV. This is not surprising, as there is high genetic homology (96.3%) between smallpox (variola) viruses and MPXV (20). Several non-clinical animal studies and a few clinical studies have shown that at least the US-licensed second-generation ACAM2000^R^, based on the first-generation vaccine “Dryvax and third-generation JYNNEOS™, also known as Imvamune or Imvanex, based on the replication-deficient MVA (Modified Vaccinia Strain Ankara) smallpox vaccines can also be used to prevent MPXV infections and/or mitigate the clinical disease (21–23). A more recent publication found low levels of MPXV-neutralizing antibodies in individuals vaccinated with the third generation MVA-based smallpox vaccine (24). This finding is supported by the observation that eight vaccinia virus proteins are targeted by neutralizing antibodies and share high sequence similarity with MPXV (25). For this reason, vaccinia-based smallpox vaccines, along with some antivirals such as Tecovirimat and Brincidofovir (26), are among the most promising tools for the prevention and control of mpox, especially in high-risk groups. The immunogenicity of *Orthopoxovirus* vaccines has traditionally been measured in terms of the presence and quantification of both binding and neutralizing anti-VACV antibodies. Antibodies induced by historic smallpox vaccination seem to induce life-long immunity (27). Now, especially after the pandemic caused by the Severe Acute Respiratory Syndrome Coronavirus (SARS-CoV-2), the Micro-neutralization (MN) assay, and Neutralization Assays (NAs) in general, are considered to be the gold standard for evaluating the immune response after natural infection and/or vaccination. In addition, since the level of neutralizing antibodies is considered to be the best laboratory predictor of protective immunity following *Orthopoxivirus* infection in humans (28), several studies have aimed to evaluate a possible surrogate of protection based on this response following vaccination/infection (29, 30). In the present study, we assessed a possible approach to performing MPXV and VACV live-virus MN assays in which the read-out is based on the production of cytopathic effect (CPE) in the cell monolayer. Given the complexity of poxviruses, the MN assay was performed in such a way as to uncover a potential role of complement, with and without the addition of an external source of Baby Rabbit Complement (BRC). It has been demonstrated with depletion experiments that complement factors are involved in EEV neutralization *in vitro* and that complement activity is required *in vivo* for strong protection. Depletion experiments showed reduction in EEV neutralization when C3 complement portion is removed. These experiments suggest that the primary mechanism of neutralization is the opsonization (31). In addition, Lusting et al. (32) proposed a membrane-lysis model of EEV neutralization where the addition of the complement provoked the lysis of the EEV membrane. A set of human serum samples from individuals who had been naturally infected with MPXV and individuals who may have and not have undergone VACV vaccinations, was used to evaluate the performance, sensitivity, and specificity of the assay.

## 2. Materials and methods

### 2.1. Serum samples and animal complement

A total of 50 human serum samples from general population (named population samples) were selected and stratified by age. It was assumed that subjects born in or before 1975 had been routinely vaccinated against smallpox according to the Italian immunization schedule (33), while subjects born in 1979 or later had not been vaccinated against smallpox. These samples were anonymously collected in 2022 in the Apulia region (Southern Italy) as residual samples for unknown diagnostic purposes and stored at the University of Siena, Italy, in compliance with Italian ethics laws. For each sample, only the date of collection and the subject’s age and sex were recorded. Written informed consent was obtained from all subjects. These samples were named using the age of individual as identifier; when individuals shared the same age, a letter was used to distinguish two samples (e.g., 85a and 85b). Two human samples from MPXV-positive patients were provided by the Department of Infectious Diseases, San Donato Hospital, Arezzo, Italy 1 month after the onset of symptoms. These samples were named ConvA 1 and ConvA 2. Two additional human samples were provided by the National Institute of Biological Standard and Controls – NIBSC (UK) as part of an international study in which VisMederi took part. The sample named NIBSC 1 is the working standard for anti-MPXV (NIBSC Code:22/218) derived from a pool of convalescent donors, while NIBSC 2 is a negative human sample. As an additional negative control, a commercial human serum sample, defined as “normal” (Lot: 3826007 Sigma, St. Louis, MO, United States), was used. Serum samples were tested in duplicate during each assay. Guinea Pig Complement (GPC) and BRC were purchased from Emozoo Srl (Casole d’Elsa, Italy) and Euroclone (Pero, Italy), respectively.

### 2.2. Cell culture, viruses, and virus growth

Vero E6 (ATCC CRL-1586) were maintained and prepared as previously reported (34). Authentic MPXV was purchased from the European Virus Archive goes Global (EVA-g SKU: 005 V-04714). VACV was acquired from the American Type Culture Collection-ATCC (ATCC Number: VR-1354™). Both viruses were propagated in Vero E6 cells according to the following protocol: 175 cm^2^ tissue-culture flasks were pre-seeded with 50 mL of Vero E6 cells (1.8×10^5^ cells/mL), diluted in Dulbecco’s Modified Eagle Medium (DMEM) (Euroclone, Pero, Italy) with 10% Fetal Bovine Serum (FBS) (Euroclone, Pero, Italy). After incubation for 18–24 h at 37°C under 5% CO_2_, flasks were washed twice with sterile Dulbecco’s phosphate buffered saline (DPBS) (Euroclone, Pero, Italy) and then inoculated with MPXV or VACV at a multiplicity of infection (MOI) of 0.003. The sub-confluent cell monolayer was incubated with the virus for 1 h at 37°C under 5% CO_2_; the flasks were then filled with 50 mL of DMEM 2% FBS and incubated at 37°C under 5% CO_2_. Cells were monitored daily until 80–90% CPE was observed. The supernatant was removed from the flasks and transferred to several falcon tubes, the cells were scraped, pooled with the supernatant, and centrifuged at 469 g for 5 min at 4°C to separate cell debris from the viral solution. The supernatant was collected, the cell pellet was resuspended in 1 mL of the supernatant and three freeze–thaw cycles were performed by placing the vial with the cell pellet on dry-ice; it was then transferred to a 37°C water bath and vortexed (all steps were carried out for 30 s) (35). We then carried out a final centrifugation at 469 g for 5 min at 4°C; the cell pellet was discarded, and the virus solution was aliquoted and stored at −80°C.

### 2.3. Micro-neutralization assay

The MN assay was performed as previously reported by Manenti et al., with minor modifications (34). Serum samples were heat-inactivated for 1 h at 56°C prior to testing. Two-fold serial dilutions, from 1:8 up to 1:4096, were then mixed with an equal volume of MPXV and VACV viral solutions containing 25 50% Tissue Culture Infectious Dose (TCID_50_) (36). The serum-virus mixture was incubated for 1 h at 37°C in a humidified atmosphere containing 5% CO_2_. After the incubation period, 100 μL of the serum-mixture was transferred to a Vero E6 cell-seeded plate. Plates were incubated for 5 days (MPXV) or for 4 days (VACV) at 37°C in a humidified atmosphere containing 5% CO_2_, then inspected by means of an inverted optical microscope to evaluate the presence/absence of CPE at each dilution point. The same assay method was performed once again, this time on adding a concentration of 5% of BRC to the virus solution, in order to obtain a final concentration of 2.5% after the addition of the diluted sample.

### 2.4. MPXV A35 and A29 protein-specific IgG-ELISA

The ELISA was performed as reported by Mazzini and colleagues (37). Briefly, ELISA plates were coated with 1 μg/mL of purified recombinant MPXV A29 Protein (AcroBiosystem, Boston, USA) or with 1 μg/mL of MPXV A35 protein (Sino Biological, China). Two-fold serial dilutions, starting from 1:100, were added to the coated plates, which were then incubated for 1 h at 37°C. After the washing step, 100 μL/well of Goat anti-Human IgG-Fc Horse Radish Peroxidase (HRP)-conjugated antibody (Bethyl Laboratories, Montgomery USA) was added. The plates were incubated at 37°C for 30 min; 100 μL/well of 3,3′,5,5′-Tetramethylbenzidine (TMB) substrate (Bethyl Laboratories, Montgomery, USA) was added. The plates were then incubated in the dark at room temperature for 20 min and read at 450 nm with a spectrophotometer.

### 2.5. Data analyses

All data were analyzed by means of GraphPad Prism 9.5.0 (730). The non-parametric Spearman correlation method was used to evaluate the correlation between MN data (MPXV and VACV).

## 3. Results

### 3.1. Virus growth and virus titration

Both MPXV and VACV reached high titers after propagation in cell culture. Both scraping and three freezing/thawing cycles in dry ice and water bath were necessary in order to achieve high viral titers. On skipping the above-mentioned cycles, very low viral titers were achieved (3.0 to 3.5 TCID_50_/mL) in comparison with those observed after freezing/thawing (from 6.5 to 7.0 TCID_50_/mL), confirming the ability of many viral particles to remain trapped within the cellular debris. The read-out time for the viral titration and, consequently, for the MN assay was evaluated on the basis of complete development of CPE in virus-positive wells. We inspected infected plates up to 6 days post-infection to evaluate any virus titer increase over time. As no substantial increase in the TCID_50_ values of MPXV and VACV and no further progression of CPE was observed after 5 days of incubation for MPXV and 4 days for VACV, the read-out of the assay was set at 5 and 4 days, respectively.

**Figure 1:**
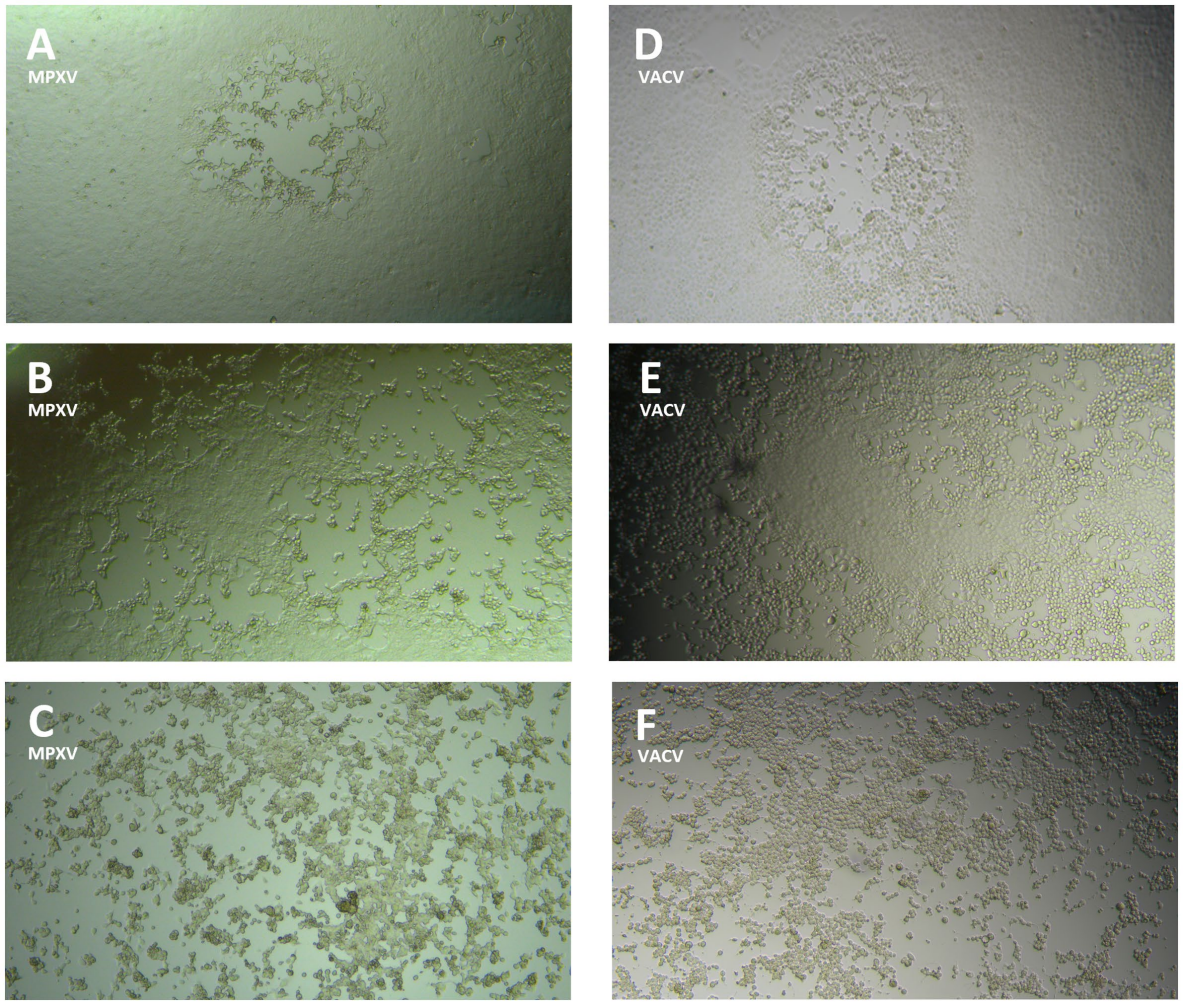
Monkeypox virus (MPXV) and Vaccinia virus (VACV) cytopathic effect (CPE) progression on Vero E6 cell monolayer: **(A)** MPXV CPE (<50%) 1–2  days post-infection; **(B)** MPXV CPE (>50%) 3 days post-infection; **(C)** MPXV CPE (100%) 4  days post-infection; **(D)** VACV CPE (<50%) 1–2  days post-infection; **(E)** VACV CPE (>50%) 3  days post-infection; **(F)** VACV CPE (100%) 4  days post-infection.

Figure 1 shows gradual progression of CPE, for both MPXV (Figures 1A–C) and VACV (Figures 1D–F), at 1–2, 3-, and 4-days post-infection, with complete destruction of the cell monolayer (corresponding to 100% CPE) on the day of the read-out.

### 3.2. Selection of complement source and concentration

Two different sources of exogenous complement were evaluated in the MN assay: BRC and GPC. Several concentrations between 2 and 5% were tested on 4 heat-inactivated serum samples: 2 mpox convalescents, 1 sample from a 60-year-old subject, and 1 negative control. Along with the serum samples, virus back-titration was performed in order to determine whether the complement concentration could interfere by reducing viral infectivity.

Concentrations above 4% were discarded, since the high BRC and GPC concentrations interfered negatively with viral infectivity, reducing the titers of the back-titration below the acceptability threshold.

**Figure 2:**
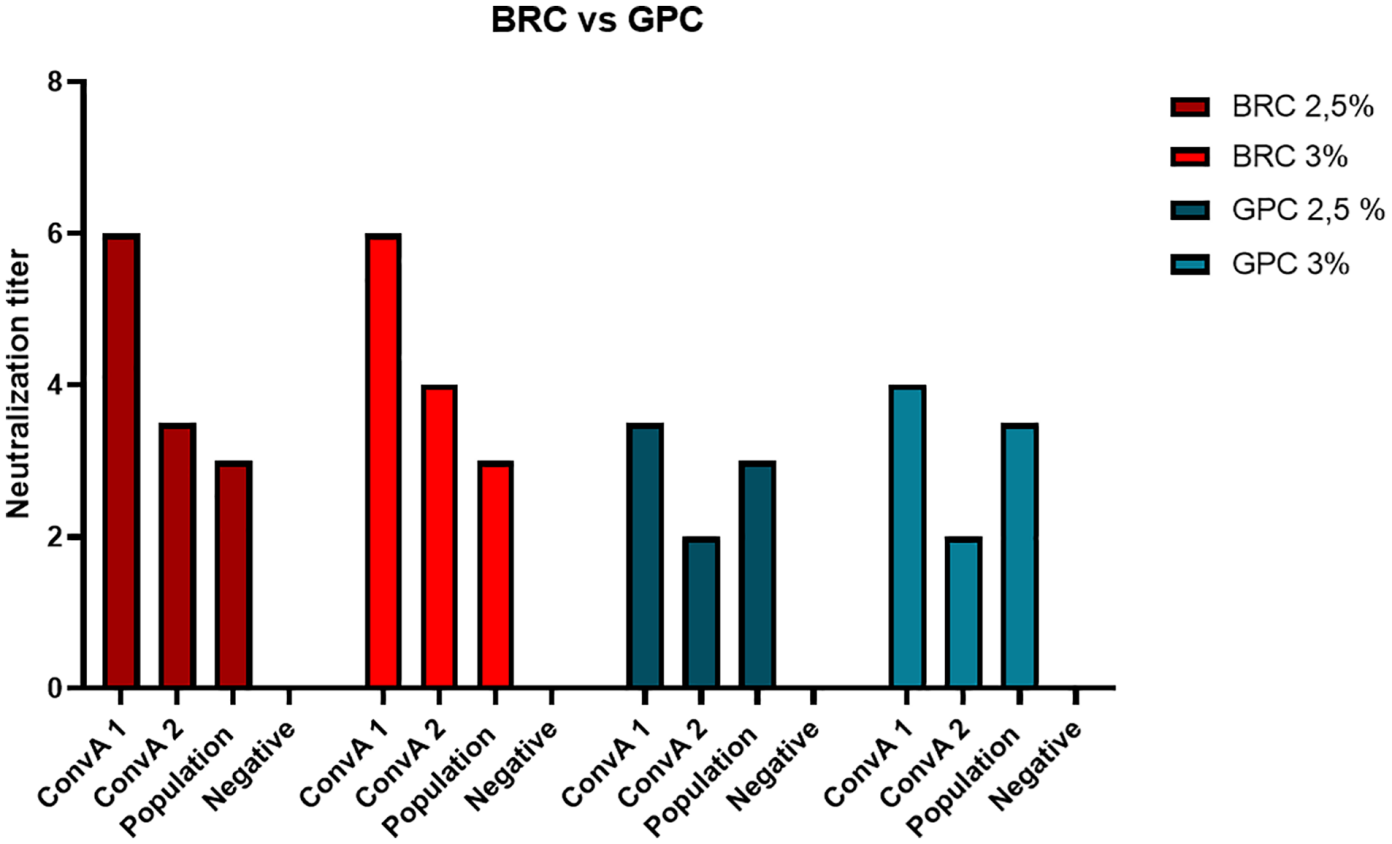
Neutralization titer achieved on using two different sources of exogenous complement, Baby Rabbit Complement (BRC) and Guinea Pig Complement (GPC), in 2 samples from mpox convalescent donors (ConvA 1 - 2), a 60-year-old subject (from population samples) and a negative human serum sample.

Figure 2 shows that 2.5 and 3% were the best concentrations for both complement sources, BRC and GPC. Moreover, we registered a greater increase in antibody titers on using BRC, indicating higher sensitivity.

Therefore, a final concentration of 2.5% of BRC was chosen as an optimal balance between sensitivity and virus titer robustness.

### 3.3. Monkeypox neutralizing results with and without baby rabbit complement

**Figure 3:**
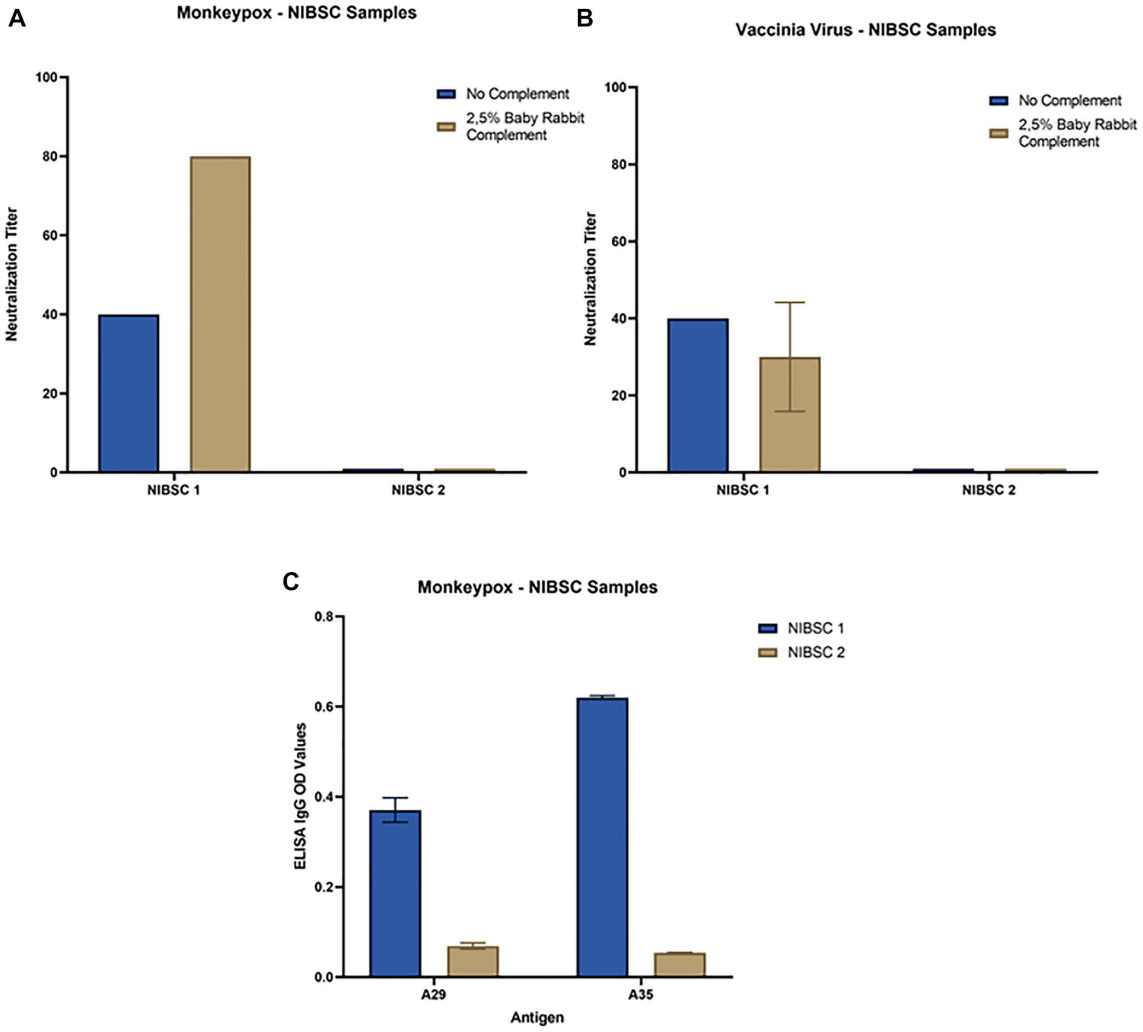
Neutralization and ELISA results for NIBSC 1–2 samples: **(A)** Monkeypox virus neutralization titers with and without Baby Rabbit Complement; **(B)** Vaccinia virus neutralization titers with and without Baby Rabbit Complement; **(C)** Monkeypox virus ELISA-IgG results for A29 and A35 surface antigens. The ELISA OD reported in the graph is referred to 1:100 dilution.

**Figure 4:**
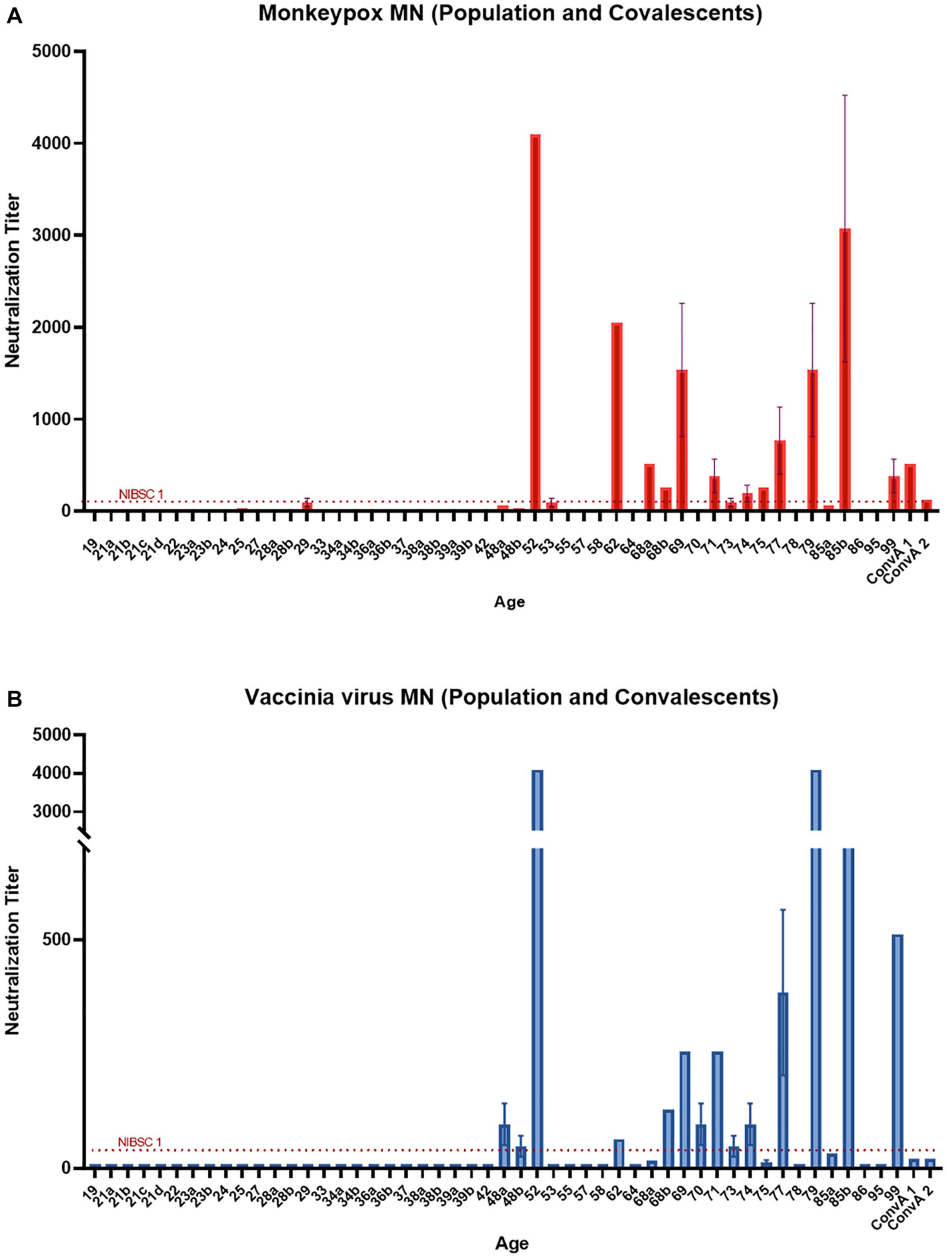
Monkeypox virus (MPXV) and Vaccinia virus (VACV) neutralization results in the presence of 2.5% Baby rabbit complement: **(A)** MPXV results for population samples and convalescent donors from San Donato Hospital (ConvA 1–2); **(B)** VACV results for population samples and convalescent donors from San Donato Hospital (ConvA 1–2). Population samples are named and reported on the *x*-axis on the basis of age.

All serum samples were tested in the MN assay with and without an external source of BRC. The samples NIBSC 1 and ConvA 1 showed detectable neutralizing titers against MPXV, with (Figures 3A, 4A, respectively) and without complement (Figure 3A for NIBSC 1, data not shown for ConvA 1). Sample ConvA 2 showed neutralization only in the presence of 2.5% complement (Figure 4A, data without complement are not shown); as expected, no neutralization was detected in NIBSC 2 or the normal human serum sample, as these were negative controls. All population samples had been taken from people who were either non-vaccinated or probably vaccinated against smallpox, but with no records of the vaccine type and number of doses; all these samples yielded negative results when tested in the MN without BRC (data not shown). However, the majority of samples from people who, according to their age, should have been vaccinated against smallpox showed variable and detectable neutralizing antibodies in the presence of BRC (Figure 4A). Specifically, in samples from subjects aged 52, 62, 69, 77, 79, and 85b, the complement-based neutralization titers observed were particularly high, indicating high antibody cross-reactions.

### 3.4. Vaccinia virus neutralization results correlated with Monkeypox neutralization results

Samples tested against MPXV were also tested against VACV in both MN assays, with and without complement. Again, in population samples, neutralization was only observed in the complement-based neutralization assay (Figure 4B). In accordance with the MPXV MN results, high neutralization titers were measured in samples from subjects aged 52, 62, 69, 77, 79 and 85b. In addition, high MN titers were also observed in subjects aged 68b, 70 and 74. Interestingly, only samples from mpox convalescent donors (NIBSC 1 and ConvA 1–2) (Figures 3B, 4B) proved positive on VACV MN assay without BRC, although the titers were quite low. Overall, a good correlation was seen in the complement-based neutralization assay against MPXV and VACV. Indeed, according to the non-parametric Spearman correlation, there was a significant positive correlation (*p* < 0.0001; *r* = 0.82) between the MPXV and VACV neutralization titers.

### 3.5. MPXV A29 and A35 protein binding activity measured By IgG ELISA

**Figure 5:**
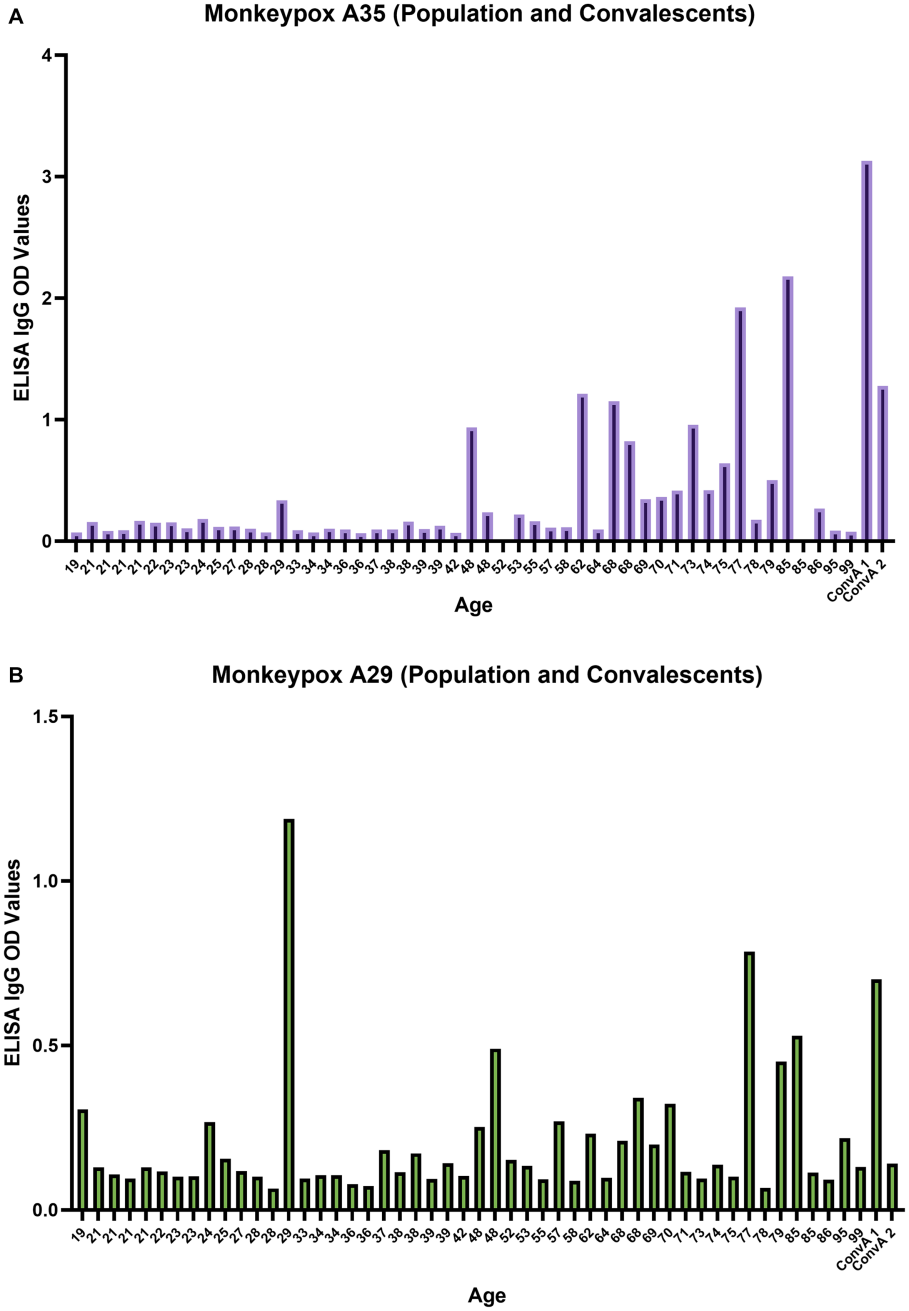
Monkeypox virus A35 and A29 antigen ELISA; **(A)** A35 antigen results for population samples and convalescent donors from San Donato Hospital (ConvA 1 - 2); **(B)** A29 antigen results for population samples and convalescent donors from San Donato Hospital (ConvA 1 - 2). Population samples are named and reported on the *x* axis on the basis of age. The ELISA OD reported in the graph is referred to 1:100 dilution.

Each serum sample was tested by means of MPXV A29 and A35 ELISA to investigate the presence of binding antibodies against the two MPXV surface proteins A29 and A35, as for NIBSC 1–2 samples (Figure 3C). As can be seen from Figure 5A there was a clear increase in anti-A35 antibodies in population samples depending on the age of the subject, demonstrating that people who had previously been vaccinated against smallpox still presented circulating binding antibodies which cross-reacted with the MPXV proteins A29 and A35. However, the detection of antibodies against A29 (Figure 5B) and their age-dependence were less pronounced than in the case of A35, although an increasing trend the signals was noted in older subjects. Moreover, on dissecting all the results for each serum sample, we observed that subjects aged from 60 years to 85 years presented quite high A35 binding signals, as well as A29. Of particular interest is the population subject 29 (29 years of age) who presented low, but detectable, MPXV neutralization activity, no anti-VACV neutralizing antibodies, detectable anti-A35 antibodies and high anti-A29 antibodies. Since we had no information on each subject’s nationality, travel history, possible exposure or previous disease/vaccination, it is difficult to postulate any explanation for this pattern; however, the concomitance of binding and neutralizing antibodies clearly shows a strong immune response against MPXV.

## 4. Discussion

The complete eradication of smallpox, which was declared by the WHO in 1980 (38), was achieved after a 10-year global effort involving thousands of health workers worldwide, who administered half a billion vaccinations. Since then, the main question facing the scientific community has been whether another *Orthopoxvirus* closely related to the human variola (smallpox) virus, such as the closely related MPXV, could fill the newly vacant ecological niche. This concern increased in 2003, after a cluster of human mpox cases was reported, caused by contact with infected prairie dogs, most likely through exposure to at least one species of rodent recently imported into the US from West Africa (39). Subsequently, a new level of threat emerged in early/mid-2022, when a unprecedented number of MPXV human infections occurred outside the endemic areas of Africa (40) which prompted the WHO to declare MPXV a PHEIC in July 2022 (15). An additional concern was raised by the discontinuation of smallpox vaccination after 1980, when the disease was declared to have been eradicated. Indeed, immune responses diminished in older vaccinated subjects, while younger generations, who had not been vaccinated against smallpox at all, had no immunity to smallpox and other zoonotic *Orthopoxvirus* infections. During the recent pandemic caused by a new human Coronavirus, named SARS-CoV-2, stringent preventive measures, such as quarantine, lockdowns, social distancing and the extensive use of masks, were implemented. The subsequent relaxation of these measures may have played an important role in spreading MPXV among humans. Even though the actual threat to humans is currently relatively low, attention must remain high. Vaccination with licensed live-attenuated non-replicating third-generation smallpox vaccines (41–43) is only recommended for high-risk groups, such as scientists and lab technicians, who may work with high titre virus, healthcare workers in general, or MPXV-exposed sexual partners (23, 43, 44). Additional research on MPXV-homologous vaccines and dedicated serological testing is needed, as is diligent investigation into the transmission and epidemiology of MPXV. In particular, the lesson learned from the SARS-CoV-2 pandemic is that we need to be prepared to face new potential public health emergencies. This means that a complete set of countermeasures, including vaccines, antiviral drugs, serological assays, diagnostic tools, public health policies, preventive practices, political awareness, and global collaborations must already be in place. Serological assays, such as the MN and ELISA tests described here, may help us to understand, and evaluate in advance, the percentage of different population groups susceptible to infections by newly emerging viruses or well-known zoonotic viruses which may cross species barriers. In a best-case scenario, such newly developed assays may allow the determination of potential surrogate markers and/or correlates of protection after natural infection and/or vaccination (45). The observation made in recent years have emphasized the importance of serological assays that are capable of detecting functional antibodies, specifically neutralizing antibodies, which can often be used for the determination of immune correlates (30).

In the present study, we started our evaluation of the performance of the MN assay on the basis of the inhibition of CPE after the incubation of different serial dilutions of human serum samples along with a standardized dose of live MPXV and VACV; although both of these viruses have quite a long history, their induction of protective immune responses has been insufficiently evaluated. Since it has been reported that many antibodies against the MPXV surface antigens are able to neutralize the virus in a complement-dependent manner, we decided to apply the MN assay with and without an external source of complement. We evaluated the performance of the assay on a panel of samples comprising MPXV convalescent serum samples, historic smallpox-vaccinated serum samples, and non-vaccinated, non-infected human serum samples. In addition, we evaluated the presence of binding antibodies against two MPXV antigens that are reported to be targets of neutralizing antibodies and involved in an important stage of the infection cycle of the virus (46). Some previous studies have demonstrated that VACV-elicited antibodies are able to cross-react with MPXV, and with orthopoxviruses in general, thereby providing some degree of protection (47).

Our results clearly show that the presence of an external source of complement not only increases the neutralization titers in samples from mpox convalescent donors but is also able to detect positive responses in samples from vaccinated subjects who have previously tested negative for both MPXV and VACV on the classical MN assay without BRC. The use of complement source not only improved the sensitivity of the assay, but also offers a better correlate of protection, mimicking the host immune response and providing strong protection *in vivo* models (31). All samples from people who, according to their age, had presumably received the VACV vaccine were tested negative on the MN assay without complement; this could indicate that the amount of “fully self-neutralizing” antibodies was low, since the vaccine had been administered many years earlier. Moreover, a couple of epitopes that may be recognized by the immune system, and which are involved in infection by MPXV (or VACV) have not yet been fully defined. In addition, different levels of antibodies are required in order to neutralize the two different forms (IMV and EEV) of the virus, and differences in pH may contribute to changing the neutralization performance (48).

To conclude, our results are in line with previously published data (24, 25) confirming that historic vaccination against smallpox is able to generate antibodies that cross-react with MPXV. However, according to our results, the presence of an external source of complement has the potential to increase the sensitivity of the assay in detecting neutralizing antibodies. In addition, antibodies elicited directly by MPXV infection are able to neutralize MPXV and to cross-neutralize VACV.

The present study has some limitations, in that a relatively small number of samples were tested and some important information was lacking, such as the exact type of vaccine administered and/or the subjects’ travel history.

The aim of this study was to evaluate the performance of a new serological (live virus-based) assay able to successfully assess the immune response induced by vaccination and natural infection in the presence of external complement, which seems to be required for efficient neutralization. Along with the ELISA, neutralization assays in general should always be included in immunogenicity studies. Although the need for BSL3 containment could be seen as a limiting factor, these types of assays are currently the only ones that can generate data on neutralizing antibodies, which are considered the best laboratory predictors of protective immunity and should be further evaluated in order to understand correlates of protection against the MPXV-induced disease and its transmissibility. Moreover, we showed that the antibodies induced by smallpox vaccination were long-lived and cross-reactive against MPXV.

## Data availability statement

The original contributions presented in the study are included in the article/supplementary material, further inquiries can be directed to the corresponding author.

## Ethics statement

Ethical review and approval was not required for the study on human participants in accordance with the local legislation and institutional requirements. The patients/participants provided their written informed consent to participate in this study.

## Author contributions

AM: conceptualization, formal analysis, writing – original draft preparation, and visualization. AM, NS, PC, and LM: methodology. NS, PC, LM, MB, LB, ML, GP, and SM: investigation. AM, SM, and GP: data analysis. NS, PC, ML, MB, LB, CS, DT, GM, SM, GP, OK, EM, and CT: writing – review and editing. CT: supervision. AM and CT: project administration. EM: funding acquisition. All authors contributed to the article and approved the submitted version.

## Conflict of interest

AM, NS, PC, LM, GP, and MB were employed by VisMederi srl. LB and ML were employed by VisMederi Research srl. EM was founder and chief scientific officer of VisMederi srl and VisMederi Research srl. CT was an external consultant of VisMederi Research srl.

The remaining authors declare that the research was conducted in the absence of any commercial or financial relationships that could be construed as a potential conflict of interest.

## Publisher’s note

All claims expressed in this article are solely those of the authors and do not necessarily represent those of their affiliated organizations, or those of the publisher, the editors and the reviewers. Any product that may be evaluated in this article, or claim that may be made by its manufacturer, is not guaranteed or endorsed by the publisher.
